# Pulmonary Vascular Sequelae of Palliated Single Ventricle Circulation: Arteriovenous Malformations and Aortopulmonary Collaterals

**DOI:** 10.3390/jcdd9090309

**Published:** 2022-09-17

**Authors:** Andrew D. Spearman, Salil Ginde

**Affiliations:** 1 Department of Pediatrics, Division of Cardiology, Medical College of Wisconsin, Children’s Wisconsin, Herma Heart Institute, 9000 West Wisconsin Avenue, Milwaukee, WI 53226, USA; 2 Cardiovascular Center, Medical College of Wisconsin, 8701 West Watertown Plank Road, Milwaukee, WI 53226, USA; 3 Department of Medicine, Division of Cardiology, Medical College of Wisconsin, 8701 West Watertown Plank, Milwaukee, WI 53226, USA

**Keywords:** congenital heart disease, single ventricle, pulmonary arteriovenous malformations, hepatic factor, aortopulmonary collaterals, Glenn, Fontan

## Abstract

Children and adults with single ventricle congenital heart disease (CHD) develop many sequelae during staged surgical palliation. Universal pulmonary vascular sequelae in this patient population include two inter-related but distinct complications: pulmonary arteriovenous malformations (PAVMs) and aortopulmonary collaterals (APCs). This review highlights what is known and unknown about these vascular sequelae focusing on diagnostic testing, pathophysiology, and areas in need of further research.

## 1. Introduction

Children and adults with single ventricle congenital heart disease (CHD) develop many sequelae during staged surgical palliation. Due to the unique single ventricle physiology, essentially all organ systems are adversely affected. The vascular system, which includes both the pulmonary and systemic vasculature, is profoundly impacted but specific adaptations to single ventricle circulation are not well understood. Previous reviews have described the unique physiology of palliated single ventricle circulation, importance of low pulmonary vascular resistance, and potential role of pulmonary vasodilators [1,2,3,4]. In this review, we will focus on pulmonary vascular remodeling and two related but distinct vascular sequelae of palliated single ventricle circulation: pulmonary arteriovenous malformations (PAVMs) and aortopulmonary collaterals (APCs). We will outline basic characteristics, clinical assessment, and key previous studies for both pathologies. Finally, we will highlight components of these sequelae that are in need of further research and potential future directions.

## 2. Pulmonary Blood Flow in Single Ventricle Circulation: Basics

Pulmonary vascular blood flow in normal biventricular circulation is characterized by low-pressure pulsatile blood flow of fully mixed systemic venous blood. Mixed venous blood in the right atrium consists of blood draining from the superior vena cava (SVC), inferior vena cava (IVC), hepatic veins (HV), and coronary sinus. Additionally, SVC blood includes lymphatic drainage from the thoracic duct, which typically drains into the innominate vein. In contrast, pulmonary vascular blood flow in surgically palliated single ventricle circulation is non-pulsatile and the mixed venous blood perfusate varies by surgical stage. After superior cavopulmonary connection (or Glenn palliation), SVC blood is the sole source of pulmonary blood flow. After total cavopulmonary connection (or Fontan palliation), venous blood from the SVC, IVC, and HV provide pulmonary blood flow.

Previous studies demonstrate that pulmonary blood flow in palliated single ventricle CHD with cavopulmonary connection is characterized by decreased flow velocity and nearly absent pulsatility [5,6]. Intravascular flow velocity contributes to wall shear stress, which is the frictional force of blood flow on the endothelium and inner vascular wall. Normal adolescents have a mean wall shear stress of ~ 5–8 dynes/cm^2^ in the branch pulmonary arteries with pulsatility increasing the maximum systolic wall shear stress up to ~15–20 dynes/cm^2^ [5,6,7]. In contrast, adolescents with palliated single ventricle circulation have a mean wall shear stress of ~3 dynes/cm^2^ and a maximum wall shear stress of only ~5–6 dynes/cm^2^, owing to the lack of pulsatile flow [5,6,7]. The significance of this decrease in shear stress and pulsatility is unknown.

In summary, pulmonary blood flow in single ventricle circulation differs drastically from normal pulmonary blood flow. Key differences that likely influence pulmonary vascular remodeling include exclusion of hepatic venous blood from the pulmonary vascular bed during Glenn palliation, chronic non-pulsatile pulmonary blood flow, and decreased velocity pulmonary blood flow altering the mechanical shear stress force on the vasculature.

## 3. Pulmonary Arteriovenous Malformations

### 3.1. Basics

PAVMs are abnormal vascular connections in the lungs between arteries and veins that cause intrapulmonary right to left shunting and hypoxia. Diffuse microscopic PAVMs develop in 60–100% of children after Glenn palliation for single ventricle CHD, regardless of the specific underlying CHD subtype (e.g., hypoplastic left heart syndrome, tricuspid atresia, etc.). Despite the universal presence of PAVMs, there are no known medical therapies to treat PAVMs [8,9]. Based on clinical observations, the untested hypothesis of the field is that hepatocytes normally secrete an unidentified factor into hepatic vein blood (called a hepatic factor) to actively regulate lung microvascular homeostasis and prevent PAVMs [10]. After single ventricle Glenn palliation, HV blood is diverted away from the lung vasculature and PAVMs subsequently develop. When a hepatic factor is re-introduced to the pulmonary circulation (i.e., after Fontan palliation or heart transplantation), PAVMs gradually resolve [11,12,13,14,15]. The field presumes that hepatic factor is still produced by the liver after Glenn palliation, but the surgically modified circulation prevents hepatic factor from reaching the lung microvasculature to inhibit PAVMs. Unfortunately, the identity, source, and mechanism of a hepatic factor remain unknown.

### 3.2. Clinical Assessment

Multiple modalities have historically been used to assess the presence of PAVMs, including peripheral oxygen saturations (SpO_2_), cardiac catheterization data (direct pulmonary vein saturation [PvO_2_], qualitative angiographic appearance of the lung parenchyma, timing of angiographic flow through the pulmonary vasculature), radionuclide lung perfusion scans, and contrast echocardiography (bubble echocardiography). A combination of several of these modalities is currently used in the clinical assessment of PAVMs; however, there is no clear consensus or guideline for assessing CHD-associated PAVMs. There are guidelines for grading bubble echocardiograms and assessing PAVMs in patients with hereditary hemorrhagic telangiectasia (HHT), which is an autosomal dominant condition associated with AVMs in multiple vascular beds [16]. This grading system for bubble echocardiograms is often extrapolated to patients with single ventricle circulation, but it is unknown if this grading is accurate or appropriate for grading CHD-associated PAVMs.

The earliest published observations of CHD-associated PAVMs were based on the combination of multiple modalities, particularly oximetry and angiography but also occasionally radionuclide lung perfusion scans [17,18]. In 1973, Mathur et al. observed rapid opacification of pulmonary veins (particularly lower > upper veins) after contrast administration, pulmonary vein desaturation (lower > upper veins), and an abnormally small number of radionuclide particles present in the right lung compared to the left lung after a lung perfusion scan in a patient with classic Glenn circulation (SVC-right pulmonary artery [RPA] anastomosis) [17]. They concluded that “most of the caval blood entering the right lung passes into the lower lobe and through widely dilated arteriovenous connections into the pulmonary vein without passing through the capillary circulation.” Similarly, McFaul et al. described, in 1977, a case series of four patients who developed PAVMs after a classic Glenn [18]. They initially identified the PAVMs using angiography and oximetry; however, they also complemented their catheterization data with echocardiography. They used M-mode echocardiograms to detect abnormal shunting into the left ventricle after injecting indocyanine green dye as a contrast agent into the RPA. In fact, the authors added an addendum to their manuscript describing their observations in two additional patients with Glenn circulation. In these two patients, they observed abnormal shunting into the left ventricle using M-mode echo and indocyanine green, but pulmonary angiography appeared normal. They made the novel conclusion that abnormal arteriovenous shunting may occur before obvious angiographic changes and that echocardiography can sensitively detect this abnormal shunting.

Based on the observation that contrast echocardiography can detect PAVMs not seen by angiography, subsequent studies began to compare echo to other modalities using agitated saline (“bubble”) echocardiography [19]. In a small and heterogeneous cohort of patients with single ventricle circulation, Chang et al. identified positive bubble studies in 10/14 patients (71%) compared to just 3/14 patients (21%) with positive pulmonary angiograms [20]. This indicated that bubble contrast echocardiography is more sensitive for detecting CHD-associated PAVMs than angiography. Similarly, Larsson et al. reported that bubble echocardiography is more sensitive for detecting PAVMs than pulmonary angiography and is associated with oxygen saturations [21]. In 20 patients with Fontan circulation, bubble echocardiography detected PAVMs more frequently (9/20, 45%) than pulmonary angiography (2/20, 10%). Additionally, those patients with positive bubble echos had lower arterial oxygen saturation at rest (88 vs 95%, *p* < 0.01) and during exercise (78 vs 89%, *p* = 0.01). In contrast, Feinstein et al. retrospectively compared bubble echo findings with cardiac catheterization data (oximetry and angiography) in 27 patients with Glenn circulation and 19 biventricular controls [22]. They reported a similar rate of detected PAVMs with echo (68/99 lungs, 69%) and angiography (65/98 lungs, 66%), but pulmonary vein desaturation (<94%) was only present in 13/45 lungs (29%). Interestingly, only 10/38 lungs with an abnormal bubble echo had pulmonary vein desaturation, and the degree of desaturation did not correlate with the severity of bubble echo findings. Despite the lack of correlation between bubble echo and oximetry, only 1/19 (5%) of bubble echos in the biventricular control group was weakly positive, indicating a low false positive rate.

Radionuclide lung perfusion scans are rarely used in current practice to assess the presence or severity of CHD-associated PAVMs; however, a previous study using lung perfusion in patients with Glenn circulation shed light on the prevalence of PAVMs [9]. Vettukattil et al. used ^99m^technetium labelled albumin microspheres to quantitatively assess intrapulmonary shunting in 17 patients with Glenn circulation, and five patients with biventricular anatomy. They reported that all patients with Glenn circulation had increased intrapulmonary right to left shunting (26.8 ± 16.9%; range 10–64%) compared to biventricular controls (5.4 ± 2.3%; range 3–8%); *p* < 0.001. Shunting was further increased in patients with Glenn circulation without additional sources of pulmonary blood flow (34.9 ± 15.8%; range 19–64%) compared to those with Glenn circulation and an additional source of pulmonary blood flow (12.0 ± 2.6%; range 10–17%); *p* < 0.001. They concluded that abnormally increased intrapulmonary shunting may be a universal finding in patients with Glenn circulation but augmenting Glenn circulation with additional pulmonary blood flow (i.e., native antegrade pulmonary blood flow containing hepatic factor) may help minimize PAVM formation.

In summary, multiple modalities can be used to assess for CHD-associated PAVMs. Bubble echocardiography is commonly used and highly sensitive but less specific for clinically significant intrapulmonary shunting. Cardiac catheterization-based data (angiography and oximetry) is less sensitive but more specific for clinically significant intrapulmonary shunting. Additional and longitudinal studies are needed to better understand the correlation between these modalities and their long-term clinical significance.

### 3.3. Previous Research: Hepatic Factor Candidates

To our knowledge, the first published use of the term “hepatic factor” was in 1993 by Dr. Richard Jonas [10]. Over the past three decades, multiple individuals and groups have proposed hepatic factor candidates and a few groups have attempted to identify this elusive factor. Most candidate factors are single proteins circulating in the blood that are believed to originate from hepatocytes, the predominant cell type in the liver. This overall hypothesis originates from early work that sought to identify hepatic factors by studying conditioned media from rat hepatocytes [23]. Unfortunately, after this initially published abstract, Marshall et al. did not report any putative factors or signaling pathways.

There have since been several proposed hepatic factor candidates (angiostatin, endostatin, BMP9, miRNA, and others), but most candidates lack supporting experimental data [24,25,26,27]. In 2013, Field-Ridley et al. quantified endostatin levels in children with single ventricle circulation using peripheral venous blood samples before and after Glenn or Fontan palliation [26]. They detected that endostatin statistically decreased from 4.4 to 3.3 ng/mL after Glenn palliation (*p* < 0.0001), whereas levels did not change after Fontan palliation (*p* = 0.3) and trended toward increasing after surgery for various forms of biventricular CHD (*p* = 0.07). It is unknown if a small absolute decrease in peripheral venous blood concentration is clinically significant or impacts lung vascular remodeling.

More recently, Capasso et al. investigated BMP9 and BMP10 as hepatic factor candidates [28]. They collected blood samples from multiple anatomic locations (right atrium, pulmonary artery, aorta, SVC, and IVC) in patients undergoing cardiac catheterizations prior to Fontan palliation (n = 9), as well as biventricular controls (n = 38). They found no differences in protein levels of BMP9, BMP10 proprotein, or the ratio of BMP9/10 in both patient groups (biventricular control and single ventricle Glenn circulation) at these various anatomic locations. They concluded that BMP9 and BMP10 are unlikely to be the unidentified hepatic factor.

### 3.4. Previous Research: PAVM Pathophysiology

Multiple studies over the past three decades attempted to understand the pathophysiology of CHD-associated PAVMs with basic science in vitro experiments, a histological study of patient biopsies, and animal models of Glenn circulation. These studies made important observations and identified models and signaling pathways to pursue in future research, but many questions remain unanswered.

Briefly described earlier, the earliest research into CHD-associated PAVMs investigated the effect of conditioned media from rat hepatocytes on bovine capillary ECs in vitro [23]. This initial study had a lasting impact on the field by proposing that hepatic factor originates from hepatocytes and functions to inhibit lung EC proliferation and subsequent AVM formation. Whether this is true in vivo, or even with human tissues, remains unknown. Studies of AVMs in other patient populations show variable data on whether or not proliferation is a key feature of AVM pathogenesis [29].

Subsequent studies used patient lung biopsies to examine lung histology. These studies made valuable early observations but, as expected, were limited by patient numbers with only two, eight, and thirteen patients in these studies [30,31,32]. They reported increased microvessel density in the affected lungs using von Willebrand factor (vWF) immunostaining to identify microvascular ECs as a marker of increased angiogenesis. They simultaneously reported decreased expression of another EC marker (CD31). This suggests that PAVM pathogenesis involves pulmonary vascular remodeling that is not well understood, perhaps with abnormal transition or de-differentiation of EC phenotype. Finally, in addition to these EC markers, they reported increased immunostaining of VEGF-A and its receptor, VEGFR2, which are key mediators in VEGF signaling [32]. VEGF signaling is known to increase EC angiogenesis, migration, and cell permeability, and increased VEGF signaling is associated with the AVM pathophysiology of patients with HHT. Ultimately, these biopsy studies concluded that VEGF-A may be an important mediator of angiogenesis in the lungs of patients after Glenn circulation. They also rightfully acknowledged that other factors and mechanisms may be important in the pathogenesis of CHD-associated PAVMs.

In addition to patient lung biopsies, several research groups studied CHD-associated PAVM pathophysiology using surgical animal models with a classic Glenn anastomosis [33,34,35,36,37,38,39,40]. Classic Glenn physiology creates non-pulsatile passive blood flow from the right-sided SVC to the RPA and right lung. The left lung continues to have antegrade pulsatile flow from the right ventricle through the left pulmonary artery to the left lung. These various animal models are summarized in Table 1. Collectively, they showed that classic Glenn circulation in several animal species recapitulates clinical findings of universal PAVM development after Glenn palliation. They demonstrated that abnormal intrapulmonary shunting can be detected with bubble echocardiography and is an early finding in the right lung (Glenn circulation) but intrapulmonary shunting is absent in the left lung (negative bubble studies). These models also showed that multiple signaling pathways are impacted in this remodeling process of PAVM development and progression. Using a combination of isolated pulmonary artery ECs and whole lung lysates, they showed increased VEGF gene expression, increased oxidative stress, changes to extracellular matrix remodeling, dysregulation of angiopoietin-TIE signaling, and others. It remains unknown, though, how these signaling pathways change throughout the dynamic process of PAVM formation and progression. It also remains unknown which pathways might be primarily impacted by flow (non-pulsatile versus pulsatile), decreased shear stress, and circulating factors (presence or lack of hepatic factor). For example, clinical observations suggest that hepatic factor can resolve PAVMs independent of pulsatile flow, but it’s unknown whether non-pulsatile flow or decreased shear stress in the branch pulmonary arteries increases susceptibility to PAVMs by “priming” the pulmonary vasculature. Or perhaps flow potentiates the function of the hepatic factor, similar to how flow potentiates BMP9-ALK1 signaling [41].

Finally, an important component of PAVM pathophysiology includes the timing of potential resolution. Previous studies suggest that PAVMs resolve over months after incorporating HV flow to the affected lung [11,12,13,14,15]. PAVM resolution is often monitored by increases in oxygen saturation, which appear to increase the most during the first 3–6 months post-Fontan (or post-heart transplant), but saturations continue to increase throughout the first year post-Fontan [11,12,13,14,15].

### 3.5. Emerging Data

In addition to the study by Capasso et al., several recent studies collected paired blood samples from the SVC (lacking hepatic factor) and IVC/HV (rich in hepatic factor) in attempts to characterize the differences between SVC and HV blood [42,43,44]. Using serum from a heterogenous group of patients composed of biventricular and single ventricle CHD, we incubated primary human pulmonary microvascular ECs with patient serum samples. We reported that after a short incubation (6–48 h) in vitro, HV serum increased pulmonary microvascular EC proliferation, increased angiogenesis, and inhibited apoptosis compared to SVC treatment [42]. As described in a commentary by Kavarana et al., these findings were opposite to the expected outcome given that hepatic factor is hypothesized to inhibit proliferation and decrease angiogenesis [45]. It remains unknown if these findings reflect a different stage of PAVM pathogenesis or are unrelated to in vivo physiology. Subsequently, we also observed multiple differences in circulating protein levels between the SVC and HV, including proteins involved in vascular angiogenic pathways and inflammatory pathways [43]. In this study, our group proposed soluble vascular endothelial growth factor receptor 1 (sVEGFR1) as a potential hepatic factor candidate.

At the same time, Bartoli et al. reported their compelling findings using paired blood samples from the HV/IVC and pulmonary artery [44]. They found differences in vWF metabolism, angiopoietin-1 and angiopoietin-2 levels, and in vitro angiogenesis in plasma from children with Glenn circulation compared to controls. Since the vWF-angiopoietin signaling axis has a key role in vascular homeostasis and angiodysplasias, they proposed that the vWF-angiopoietin axis may play a major role in CHD-associated PAVMs and represents a target to potentially normalize adverse remodeling in CHD-associated PAVMs. These are intriguing new findings that warrant further investigation, especially given that inhibiting angiopoietin-2 prevented and even rescued AVMs in a recently published mouse model of HHT [46].

## 4. Aortopulmonary Collaterals

### 4.1. Basics

Aortopulmonary collateral blood vessels are vascular connections between the systemic arterial system and the pulmonary vasculature. Aortopulmonary collaterals (APCs) universally develop but to varying degrees in patients with single ventricle CHD. APCs increase oxygen saturations in the short-term; however, APCs in single ventricle CHD create a hemodynamic burden by routinely shunting up to 30–50% of total systemic blood flow to the lungs [47,48,49]. Thus, APC formation is a maladaptive compensation for single ventricle circulation. Despite the universal development of APCs in patients with single ventricle CHD, many knowledge gaps remain, including the most accurate diagnostic modality for APCs, short- and long-term clinical significance of APCs, the pathophysiology of APCs, and others.

The lungs are normally perfused through two separate vascular units: the pulmonary arteries and the bronchial arteries. The pulmonary arteries supply deoxygenated blood to the lung parenchyma to participate in gas exchange and ultimately supply the body with oxygenated blood. In contrast, the bronchial arteries supply oxygenated blood to the tracheobronchial tree and large-caliber pulmonary arteries to deliver necessary oxygen to these tissues but do not generally participate in gas exchange. Ultimately, both circulations drain through the pulmonary veins and constitute total pulmonary blood. Normally, pulmonary arterial blood flow constitutes 95-99% of total pulmonary blood flow and bronchial artery blood flow provides ~1–5% [49,50,51,52]. In palliated single ventricle circulation, APCs often supply up to ~ 40–50% of pulmonary blood flow [50,53,54,55].

Bronchial arteries normally arise from the thoracic descending aorta and are described as “orthotopic bronchial arteries”, but they can also arise elsewhere from the aorta and proximal aortic branches (so-called “ectopic bronchial arteries”) [52]. These “ectopic bronchial arteries” usually arise from the transverse aortic arch, subclavian arteries, and the proximal branches of the subclavian arteries (internal mammary arteries, thyrocervical trunk, lateral thoracic arteries, etc.). In single ventricle CHD, previous work indicates that APCs predominantly arise as ectopic bronchial arteries from branches of the subclavian arteries (internal mammary arteries [34%], thyrocervical trunk [22%], thoracodorsal arteries [13%]), while the “true” or orthopic bronchial arteries are the origins for a minority (18%) of APC vessels [56]. Thus, APC blood flow in single ventricle CHD comes from both orthotopic and ectopic bronchial arteries.

Many aspects of APC pathophysiology remain unknown, including the specific stimuli for APC development, whether APCs represent arteriogenesis (i.e., angiogenic growth of new vessels from arteries) versus opening of pre-existing vascular channels (i.e., abnormal vascular dilation), or perhaps both [57]. Additionally, it’s well reported that our field does not yet have a consensus on when, how, or if we should intervene in APCs [58,59].

### 4.2. Clinical Assessment

Historically, APCs were exclusively diagnosed during cardiac catheterization using oximetry and/or angiography [60,61]. Oximetry can indirectly demonstrate significant APC shunting if there is a step-up in saturations from the proximal pulmonary arteries (or SVC/IVC) to the more distal pulmonary arteries (i.e., distal to the location of APC connection to the pulmonary arteries). Angiography provides more direct visualization of APC shunting. Pulmonary angiography may show a lack of filling in the pulmonary vascular bed where APC flow predominates, most commonly in the upper lobes. On the other hand, aortic angiography (or more direct subclavian injections) can directly fill the APCs to show abnormal connections between the arterial system and pulmonary circulation. However, these catheter-based methods are highly user-dependent, and sensitivity varies based on injection location and technique. Indeed, a recent survey of Pediatric Cardiology interventionalists identified significant variability in practices for assessing APCs during pre-Fontan catheterizations [59].

APCs can also be assessed in the operating room while patients are on cardiopulmonary bypass [47,48]. This method allows direct quantification of APC shunting. While fully supported on cardiopulmonary bypass, all systemic venous return flows through the bypass circuit and then flows through the arterial cannula to re-enter the patient’s arterial circulation. Thus, blood flow can only reach the left heart if it is shunted from the aortic flow to the pulmonary vasculature. The volume of blood returning to the left heart is then quantified as the APC burden and is expressed as a fraction or percent of the total bypass flow. Unfortunately, this technique quantifies APC flow under non-physiologic conditions and is highly invasive, so its use is limited.

In 2009, two groups introduced cardiac MRI as a novel non-invasive tool to quantify APC flow [50,53]. These landmark studies shifted the clinical assessment of single ventricle APCs such that MRI is now routinely used for quantitatively assessing APC burden. MRI is able to estimate APC flow by using phase–contrast imaging, which can estimate blood flow volumes in specific anatomic locations. There are two main approaches for estimating APC flow with MRI. One approach calculates APC flow as the difference between return and supply of pulmonary blood flow (APC flow = sum of pulmonary vein flow − sum of pulmonary artery flow). A second approach calculates APC flow as the difference between supply and return of systemic blood flow (APC flow = ascending aorta flow − [sum of SVC + IVC flow]). Unfortunately, MRI assessment of APC flow is likely underutilized because it is obtained as part of a complete cardiac MRI, and cardiac MRI requires general anesthesia and mechanical ventilation with breath holds for accurate imaging in young children.

A previous study attempted to identify whether echocardiography could serve as a non-invasive and readily available tool to assess APC flow. Di Maria et al. retrospectively investigated the correlation between descending aorta spectral Doppler and APC flow [62]. Unfortunately, they found that there was no correlation between descending aorta spectral Doppler and MRI-quantified APC blood flow (r^2^ = 0.006, *p* = 0.46). To our knowledge, no other studies have investigated the utility of ultrasound to assess APC flow, and our field continues to lack an objective bedside tool to assess APC flow.

In summary, the catheterization-based assessment of APC flow (angiography and oximetry) is widely used but is qualitative, insensitive, and highly user-dependent. MRI-based quantification of APC flow is a quantitative method for assessing APC flow that is becoming the standard of care. However, the correlation between cath and MRI for quantifying APCs is not yet well understood. Additionally, MRI-based quantification remains underutilized, likely because it is time-consuming and requires general anesthesia in young children. An objective and easily accessible modality (i.e., minimally invasive or non-invasive bedside test) could improve our clinical assessment of APCs by providing more data on the timing of APC development and the potentially dynamic nature of APC flow.

### 4.3. Previous Research: Clinical Significance of APCs

Previous studies used these different modalities to quantify APC flow, and, not surprisingly, there are mixed data about the clinical significance of APCs (Table 2) [47,48,54,60,61,63,64]. The earliest studies investigating whether APC flow impacts early Fontan outcomes used a combination of direct flow measurement on cardiopulmonary bypass and angiography during pre-Fontan catheterization. Their results were highly variable with some studies reporting that APCS are a risk factor for worse post-Fontan outcomes [47,60], another reporting no impact post-Fontan outcomes [48], and one study even reporting better post-Fontan outcomes with greater APCs [61]. In contrast, three more recent studies that all used MRI found that increased APCs are associated with adverse early post-Fontan outcomes, including increased chest tube duration and post-operative length of stay [54,55,63].

In addition to the association between APC burden and early post-Fontan outcomes, APCs can adversely impact patients in multiple ways. It’s well recognized that APCs can increase the risk of life-threatening pulmonary hemorrhage and hemoptysis, although the frequency and absolute risk of this event are not well reported [64,65]. APCs can affect long-term single ventricle circulation by chronically causing flow energy loss in palliated single ventricle circulation [66]. APCs can also decrease cerebral blood flow, which may adversely impact neurodevelopment and longer-term neurocognitive function [67]. Finally, APCs create a hemodynamic burden for patients who require mechanical circulatory support and heart transplantation [68,69,70,71].

### 4.4. Previous Research: APC Pathophysiology

Even though APCs are universal in patients with palliated single ventricle circulation, we do not yet have conclusive data on the specific factors that promote or inhibit APC formation and progression. Multiple studies investigated clinical variables that may be associated with APC development. Potential variables associated with increased APCs include small branch pulmonary arteries [47,65,72,73], female gender [74], previous BT shunt [53,56,61,72,74], previous thoracic surgery [56], and younger age at Glenn palliation [56,63,73]. The specific underlying sub-type of single ventricle CHD and the dominant ventricular morphology do not appear to be associated with APC burden [48,63,74]. When and to what degree systemic hypoxia contributes to APC development is not clearly defined. Intuitively, hypoxia is likely a trigger for APC development. Previous studies reported that higher arterial saturations are associated with the presence of APC collaterals [48,56,74], which supports that APCs can participate in gas exchange and increase oxygenation. However, the presence of APCs confounds the analysis of how hypoxia contributes to APC development, or whether there is a threshold of hypoxia that promotes APC development.

Recent MRI-based studies started to clarify the timing of APC development and potential regression [49,53,72]. These studies showed that APC flow appears to be greatest during the period of Glenn circulation and regresses to some degree after Fontan palliation. Whitehead et al. also identified that APCs may actually increase in the short-term immediately following Fontan palliation [49]. It’s unknown if this potential increase in APC flow is due to post-operative inflammation or other factors. There continues to be great variability in APC flow among patients with Fontan circulation, even 15-20 years after Fontan palliation [49,53,72].

Animal models of unilateral branch pulmonary artery ligation demonstrate that bronchial and intercostal arteries dramatically increase blood flow within days to the ipsilateral lung [75,76]. This suggests that APCs can develop quickly. A previous classic Glenn animal model investigating PAVMs also clearly observed tortuous collateral vessels (i.e., APCs) on the pleural surface of the ipsilateral lung [39]. Thus, APCs appear to hone to specific a lung, or potentially even lung segment, as opposed to a systemic response to both lungs. This localized response may be mediated by paracrine signaling from focal tissue ischemia, decreased vascular shear stress, or other mechanisms that are not yet well-defined.

## 5. Future Directions

PAVMs and APCs are universal pulmonary vascular sequelae for patients with single ventricle circulation. As described, they create unique physiologic burdens that are well recognized, but the specific pathologic factors driving their development are not well understood. To clearly define the pathophysiologic mechanisms for each condition, we need to invest in animal models and translational studies. Using previously demonstrated animal models of classic Glenn physiology can be leveraged with more advanced molecular tools that were not previously available or were financially prohibitive. For example, more price-friendly RNA-sequencing should permit a more comprehensive analysis of transcriptome data, and the emergence of single-cell RNA-sequencing can provide greater insight into the multitude of cell types that are impacted by single ventricle circulation.

Lastly, it is imperative that we continue to advance multi-institution collaborations. The inclusion of more patients with greater diversity into prospective studies is necessary for a more granular analysis of patient and clinical variables.

## 6. Summary

Single ventricle palliation drastically changes pulmonary blood flow—both the source of the blood and the means of perfusion to the lungs. PAVMs and APCs are two distinct but related processes that result from the unique physiology of patients with single ventricle CHD. Our field has recognized these processes for decades, but our understanding remains limited. Further studies are essential so we can develop medical therapies that are currently lacking.

## Figures and Tables

**Table 1:** Studies using animal models of Glenn circulation.

Study	Animal Model	Methods and Time Points	Results	Conclusions
Malhotra, Riemer, Thelitz, et al., 2001 [33]	Lamb Glenn and sham control, n = 12 each	Bubble echo at 8 weeks RNA and protein quantification (1, 2, 5, 15 weeks)	Reduced ACE activity and angiotensin II protein levels at 1, 2, and 5 weeks; no difference at 15 weeks	Early decrease in ACE and angiotensin II post-Glenn, potentially indicating decreased endothelial vasoconstriction after Glenn
Malhotra, Reddy, Thelitz, et al., 2002 [34]	Lamb Glenn and sham control, n = 12 each	RNA and protein quantification (1, 2, 5, 15 weeks)	Increased angiotensin II receptor type 1 (AT1) and type 2 (AT2) RNA and protein (at 1, 2, and 5 weeks)	AT1 and AT2 are likely involved early in pathologic vascular remodeling after Glenn
Malhotra, Reddy, Thelitz, et al., 2002 [35]	Lamb Glenn, PA band, and Sham control, n = 6 each	Bubble echo at 8 weeks Protein quantification (2 and 5 weeks)	Glenn and PA band increases angiogenic gene expression (VEGF-A, VEGFR1) Glenn (but not PA band or sham) increases stress-related genes (heme-oxygenase 1, GLUT1, HIF-1α, CD62)	Glenn circulation uniquely induces oxidative stress and may predispose to PAVMs
Starnes, Duncan, Fraga, et al., 2002 [36]	Rat * n = 8	Pulmonary angiography (2–13 months) Histology (13 months)	Increased microvessel density starting at 4–8 months Angiographic evidence of PAVMs starting at 11 months	Rat model recapitulates clinical PAVMs after Glenn
Mumtaz, Fraga, Nicholls, et al., 2005 [37]	Rat * n = 3	RT-PCR at 2, 8, and 12 months	Progressively increased VEGF-A gene expression over time	Possible role for VEGF in PAVMs
Tipps, Mumtaz, Leahy, Duncan, 2008 [38]	Rat * n = 6	RNA microarray at 8 months	Multiple affected pathways: - angiogenesis (angiopoietin-2, placental growth factor, tip cell markers) - matrix remodeling (matrix metalloproteinases, collagen subtypes) - vascular tone (endothelin 1, endothelin receptor B)	Diverse signaling pathways are altered at the 8-month time-point
McMullan, Reddy, Gottliebson, et al., 2008 [39]	Lamb * n = 40	Bubble echo (0–27 weeks) Morphologic inspection and vascular casting (2–27 weeks)	Positive bubble studies starting at 5 weeks Dilated and tortuous subpleural vessels arising from bronchial arteries (i.e., APCs) Multiple direct arteriovenous connections (minimal diameter 15–20 µm) visualized with casting	All animals develop intrapulmonary shunting on echo by 6 weeks Central PAVMs bypass alveolar capillaries and have diameter ≥ 15 µm Surface APCs develop separately from PAVMs
Kavarana, Mukherjee, Eckhouse, et al., 2013 [40]	Pig * n = 5	Bubble echo Isolated PAEC at 6–8 weeks	Positive bubble studies Comparing right PAECs vs left PAECs: Increased proliferation, tube formation, and gene expression of angiopoietin-1 and TIE2	Pig model PAVMs detectable by bubble echo Multiple changes in PAEC phenotype and gene expression

* Models used classic Glenn circulation (right-sided superior vena cava anastomosis to right pulmonary artery) with the left lung serving as control lung comparison. APCs = aortopulmonary collaterals, LPA = left pulmonary artery, PAEC = pulmonary artery endothelial cell, RPA = right pulmonary artery.

**Table 2:** Studies investigating the impact of APC flow on clinical outcomes.

Study	Method for APC Quantification	Study Size	Results	Conclusions
Ichikawa, Yagihara, Kishimoto, et al., 1995 [47]	Direct flow measurement on cardio-pulmonary bypass during Fontan palliation	n = 33	APC flow: 6–55% of total pump flow Greater APC flow associated with increased post-Fontan systemic venous pressures (*p* < 0.01)	High APC flow is a risk factor for Fontan operation
Spicer, Uzark, Moore, et al., 1996 [60]	Angiography during pre-Fontan catheterization Graded on 4-point scale	n = 71	60/71 (84.5%) patients had APCs visualized angiographically Higher APC grade is associated with more prolonged pleural chest tube drainage APC occlusion is associated with shorter duration pleural drainage	APCs increase the risk for prolonged pleural effusions post-Fontan “Significant” APCs should be occluded at pre-Fontan catheterization
McElhinney, Reddy, Tworetzky, et al., 2000 [61]	Angiography during pre-Fontan catheterization Binary classification (APCs present or absent) based on angiographic criteria	n = 76 with previous Glenn palliation	APCs were identified in 49/76 (59%) of patients after Glenn palliation and 22/43 (51%) after Fontan Duration of chest tubes post-Fontan was shorter in patients with identified APCs (8 ± 6 vs 19 ± 15 days, *p* = 0.007) Patients with APCs identified were more likely to have pulmonary arterioplasty with Fontan (67% vs 24%, *p* = 0.04)	APCs were not associated with worse post-Fontan outcomes Paradoxically, APCs identified angiographically were associated with better outcomes
Bradley, McCall, Sistino, et al., 2001 [48]	Direct flow measurement on cardio-pulmonary bypass during Fontan palliation	n = 32	APC flow: 9–49% (median 19%) of total pump flow APC flow (3 control patients): 0.5–1.4% of total pump flow Greater APC flow had no effect on post-op Fontan pressure, atrial pressure, transpulmonary gradient, duration of pleural effusions, or resource utilization post-Fontan	APCs are universal but a degree of APC flow varies widely APC flow does not impact Fontan outcomes, but results may not be generalizable to higher-risk Fontan candidates
Odenwald, Quail, Giardini, et al., 2012 [63]	Pre-Fontan MRI	n = 65	Greater APC flow associated with increased post-Fontan chest drain volume (*p* = 0.001), chest drain duration (*p* = 0.005), ICU LOS (*p* = 0.04), hospital LOS (*p* = 0.048)	Increased APC flow is strongly associated with adverse early post-Fontan outcomes independent of conventional risk factors
Glatz, Rome, Small, et al., 2012 [55]	Pre-Fontan MRI	n = 44	APC flow: 31 ± 11% (Qs), 44 ± 15% Qp APC flow associated with hospital admission ≥ 7 days (OR = 9.2, *p* = 0.02) and chest tube duration ≥ 10 days (OR = 22.7, *p* = 0.009)	Increased APC flow is associated with increased post-Fontan hospitalization and chest tube duration
Grosse-Wortmann, Drolet, Dragulescu, et al., 2012 [54]	Pre-Fontan MRI	n = 33	APC flow: 35 ± 12% (Qs), 43 ± 13% Qp Greater APC flow associated with greater duration of hospital stay (*p* = 0.02) and pleural drainage (*p* = 0.03)	Increased APC flow is associated with adverse early post-Fontan outcomes

APCs = aortopulmonary collaterals, ICU= intensive care unit, LOS= length of stay.

## Data Availability

Not applicable.
